# Peridomestic *Aedes malayensis* and *Aedes albopictus* are capable vectors of arboviruses in cities

**DOI:** 10.1371/journal.pntd.0005667

**Published:** 2017-06-26

**Authors:** Ian H. Mendenhall, Menchie Manuel, Mahesh Moorthy, Theodore T. M. Lee, Dolyce H. W. Low, Dorothée Missé, Duane J. Gubler, Brett R. Ellis, Eng Eong Ooi, Julien Pompon

**Affiliations:** 1 Program in Emerging Infectious Disease, Duke-NUS Medical School, Singapore; 2 Department of Clinical Virology, Christian Medical College, Vellore, Tamilnadu, India; 3 Department of Biological Sciences, National University of Singapore, Singapore; 4 MIVEGEC, UMR IRD 224-CNRS5290-Université de Montpellier, Montpellier, France; North Carolina State University, UNITED STATES

## Abstract

**Background:**

Dengue and chikungunya are global re-emerging mosquito-borne diseases. In Singapore, sustained vector control coupled with household improvements reduced domestic mosquito populations for the past 45 years, particularly the primary vector *Aedes aegypti*. However, while disease incidence was low for the first 30 years following vector control implementation, outbreaks have re-emerged in the past 15 years. Epidemiological observations point to the importance of peridomestic infection in areas not targeted by control programs. We investigated the role of vectors in peri-domestic areas.

**Methods:**

We carried out entomological surveys to identify the *Aedes* species present in vegetated sites in highly populated areas and determine whether mosquitoes were present in open-air areas frequented by people. We compared vector competence of *Aedes albopictus* and *Aedes malayensis* with *Ae*. *aegypti* after oral infection with sympatric dengue serotype 2 and chikungunya viruses. Mosquito saliva was tested for the presence of infectious virus particles as a surrogate for transmission following oral infection.

**Results:**

We identified *Aedes albopictus* and *Aedes malayensis* throughout Singapore and quantified their presence in forested and opened grassy areas. Both *Ae*. *albopictus* and *Ae*. *malayensis* can occupy sylvatic niches and were highly susceptible to both arboviruses. A majority of saliva of infected *Ae*. *malayensis* contained infectious particles for both viruses.

**Conclusions:**

Our study reveals the prevalence of competent vectors in peri-domestic areas, including *Ae*. *malayensis* for which we established the vector status. Epidemics can be driven by infection foci, which are epidemiologically enhanced in the context of low herd immunity, selective pressure on arbovirus transmission and the presence of infectious asymptomatic persons, all these conditions being present in Singapore. Learning from Singapore’s vector control success that reduced domestic vector populations, but has not sustainably reduced arboviral incidence, we suggest including peri-domestic vectors in the scope of vector management.

## Author summary

Dengue and chikungunya are mosquito-borne diseases and re-emerging as a global burden of the 21^st^ century. Because of the absence of cure and limitations of the current vaccine, vector control remains the sole efficient intervention to mitigate epidemics. The highly-populated city of Singapore represents an example of successful vector control, where the primary vector *Aedes aegypti* has practically disappeared from households. However, there as well, dengue and chikungunya are re-emerging. In this study, we surveyed peridomestic sites for mosquito vectors and identified *Aedes albopictus* and *Aedes malayensis* throughout Singapore and in open-air areas frequented by people. We further showed that both mosquito species are highly susceptible to dengue and chikungunya viruses and detected infectious virus particles in mosquito saliva, indicating their transmission capacity. Our study provides evidence that *Ae*. *albopictus* and *Ae*. *malayensis* possess all the traits necessary to contribute to virus transmission in cities and suggest that peridomestic areas be included in vector management programme.

## Introduction

Globally, dengue virus (DENV) is the most commonly transmitted arbovirus and infects an estimated 390 million people per year with greater than half the world’s population at risk of infection [1]. Chikungunya virus (CHIKV) periodically emerges to cause epidemics in populated areas and is maintained in sylvatic cycles [2, 3]. In domestic settings, both viruses are primarily transmitted by *Aedes aegypti* [4, 5]. Its prolific adaptation to urban environment, its near-exclusive anthropophilic blood-feeding behaviour, and its proclivity to take multiple blood meals during a single gonotrophic cycle make it the ideal domestic vector [6]. *Aedes albopictus* belongs to the same subgenus (*Stegomyia)* and is considered a secondary vector of DENV and CHIKV [2], primarily because of its catholic feeding habits and peridomestic (defined here as city parks and green corridors interspersing housing estates) biology. *Aedes albopictus* mosquitoes will utilize oviposition sites distant from human habitats [5], but prefer humans as hosts even when other vertebrates are available [7]. This species is a biting nuisance [8, 9] and will enter houses and feed indoors [10, 11]. Both mosquito species are globally distributed and their ranges are expanding [12], facilitating the increasing spread of DENV and CHIKV. With a partially efficacious vaccine against dengue [13, 14] and an absence of curative treatment for both diseases, vector control remains the sole intervention to mitigate these epidemics. However, re-emergence and recent global intensification in the frequency and scale of dengue and chikungunya outbreaks, especially in large cities, [1, 15, 16] have raised concerns about the sustainability of the current approach targeting domestic vectors [1, 16, 17].

In Singapore, dengue outbreaks first appeared in 1960 and rapidly became a major cause of childhood mortality [18]. The subsequent government implementation of a comprehensive and permanent integrated mosquito management program drastically decreased the *Aedes* house index (the percentage of houses with *Aedes* mosquitoes) and succeeded in maintaining a low disease incidence until the early 1990s [19]. Today, the vector control programme systematically applies WHO recommendations [5, 20] and includes surveillance, source reduction, reactive adulticiding, insecticide resistance monitoring, public health education, community participation, fines for allowing mosquito breeding, and georeferenced entomologic and clinical surveillance for an annual cost of US$50 million [21].Together with the wide availability of technical amenities like air-conditioning, window screens, better sanitation [22, 23] and piped water supplies [24] that reduce oviposition sites, the extensive vector control program maintains a very low *Aedes* house index [19]. Yet, dengue epidemics have increased in frequency and intensity for the past 15 years [25, 26], and chikungunya and Zika virus emerged in 2008 and 2016, respectively [27, 28].

**Fig 1:**
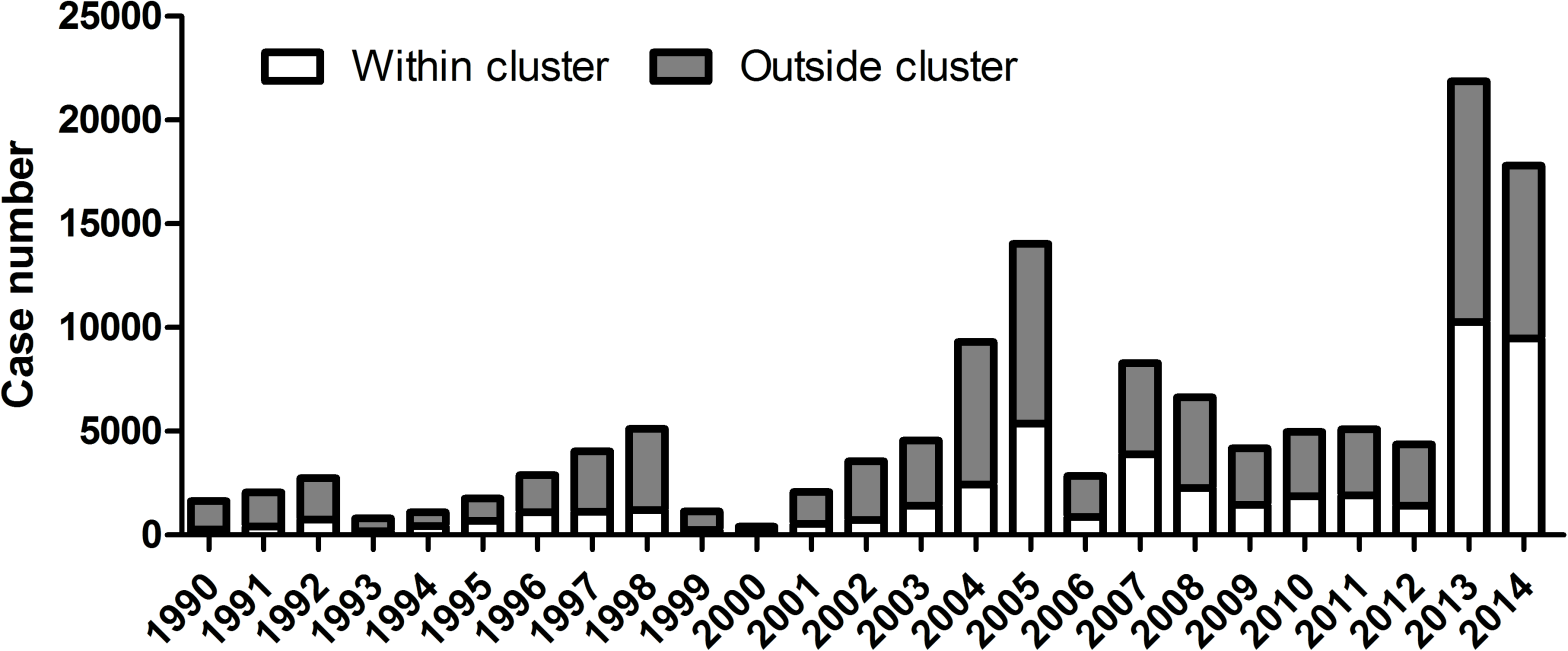
Distribution of cases identified within and outside case-clusters from 1990 to 2014. A case-cluster is defined as two or more infected persons epidemiologically linked by place [within 150m (200m till 2002)] with respect to their home address and time (within 14 days). Numbers above bars represent percentage of cases outside clusters [30].

While lowered herd immunity resulting from 30 years of low dengue incidence increased the force of infection [18, 19], there remains an apparent paradox between the very low *Aedes* house index and the resurgence of dengue and chikungunya in Singapore. To resolve this paradox, we hypothesize that foci of infection exist in peridomestic areas. Several epidemiological observations indicate that non-domestic infections are frequent in Singapore: (i) symptomatic cases are more prevalent in adults than in young children [27, 29], suggesting that the principal site of infection is not in homes where children spend most of their time, (ii) dengue incidence markedly increases for children at the age they start school and thus spend more time outside their home [29], (iii) women, who are more likely to stay home caring for children, have a lower incidence of infection than men [29], and (iv) a majority of infected patients do not live in areas of case-clusters (defined as two or more cases living within 150m from each other and occurring within 14 days), suggesting a similar proportion of infections do not occur in domestic clusters (Fig 1) [30].

The structure of Singapore is a matrix of highly populated areas with parks and bosquets, resulting in a vegetation cover of 44% [31]. Forested areas such as parks are frequented weekly by at least 35% of the population [32] and possess all social and environmental characteristics identified in foci of infection [33], i.e. routine attendance, artificial or natural containers, open spaces for daily activities, and the lack of air-conditioning. To determine the potential risk of infection in these sites, we combine country-wide entomological surveys and vector competence studies to determine the distribution and prevalence of competent vectors.

## Materials and methods

### Sampling surveys

Between 2013 and 2015, oviposition traps filled with distilled water containing a crushed wafer of fish food (TetraMin fish flakes) and a piece of seed germination paper (Anchor Paper) were placed at 12 forested sites throughout Singapore (West Coast Park, Clementi Woods Park, Mount Faber Park, Bukit Batok Town Park, East Coast Park (x 3 sites), Sentosa, Pulau Semkau, Kent Ridge Park, Whampoa along the Kalong River, and Bukit Timah Nature Reserve). Oviposition papers were replaced every 3–4 days. Upon collection, oviposition papers were treated with a 10-fold commercial bleach dilution to surface sterilize them and prevent microbial growth and introduction of field parasites into laboratory colonies. Egg papers were exposed for 10 minutes to this solution before rinsing and drying and kept between 2–4 weeks before hatching. Eggs were hatched in deoxygenated water, larvae were fed a mix of fish food (TetraMin fish flakes) and liver powder (MP Biomedicals), and adults were provided a 10% sucrose solution. The insectary was held at 28°C with 50% humidity on a 12:12h dark:light cycle. Adult mosquitoes were killed by freezing and identified using taxonomic keys to distinguish *Ae*. *albopictus*, *Ae*. *aegypti* and *Ae*. *malayensis* [34] (S1 Fig). All collections were undertaken with permission of the National Parks Board, Singapore (Permit NP/RP11-012-2).

### Presence in open-air areas

We conducted a transect study of oviposition preference at two separate sites (transect A and B) in East Coast Park (southern part of Singapore). Both sites were chosen based on the presence of adjacent forested and open(no tree cover)-air patches. In each site, we placed oviposition traps (as above) every 15m along each transect that traversed both forested and open-air patches; three traps in the forested area, and three others in the open area. Oviposition collection was undertaken as described above from 25 July to 23 September 2014 every 3–4 days for a total of 16 and 18 collection dates for transect A and B, respectively. Eggs were counted visually with an Olympus SZ-61 stereoscope and were reared to adults as described above for identification.

### Colony establishment and mosquito rearing

The *Ae*. *malayensis* colony was established in 2014 from eggs collected using oviposition traps in 2 different forested areas located in the Northern (Sembawang) and the Southern (East Coast Park) regions of Singapore (National Environment Agency Permit NEA/PH/EHD/13-00011). The *Ae*. *aegypti* and *Ae*. *albopictus* colonies were established in 2010 from eggs collected using oviposition traps from a single neighborhood (Ang Mo Kio-central area) in Singapore. Eggs were reared as described above and 500 morphologically identified adults [34] (S1 Fig) were used to start each colony. Adult mosquitoes were held in rearing cages (Bioquip) supplemented with 10% sucrose and fed pig’s blood (Primary Industries Pte Ltd) twice weekly. To maintain field genetic diversity, the *Ae*. *aegypti* and *Ae*. *albopictus* colonies were replenished every month from 2010–2014 with 40 to 100 adults obtained from the same site, while all *Ae*. *malayensis* used in the infection experiments were F3 and F4 generations.

### Virus isolates and propagation

We used the clinical isolates dengue ST (passage 6) [35] and Chikungunya EAS DMERI09/08 (passage 3) viruses, collected from the Singapore General Hospital in 1997 and National University Hospital, Singapore in 2008, respectively (already existing collection). A portion of the E1 gene from CHIKV EAS was amplified and sequenced to validate the absence of the A226V amino acid substitution [9]. All viruses were propagated in Vero cells (ATCC) and titrated by plaque assay in BHK cells (ATCC) as previously described [36].

### Oral infection and dissemination study

Three to five day-old *Ae*. *aegypti*, *Ae*. *albopictus* or *Ae*. *malayensis* females were sugar-deprived for 24 h and subsequently offered a blood meal containing a 40% volume of washed erythrocytes from SPF pig’s blood (PWG Genetics), 5% of 100 mM ATP (Thermo Scientific), 5% human serum (Sigma) and 50% volume of a DENV ST or CHIKV EAS virus in RPMI (Gibco). The final concentration of virus in the blood mix was 1x10^7^ pfu/ml and was validated by plaque assay [36]. Mosquitoes were exposed to the artificial blood meal for one hour using a Hemotek membrane feeder system (Discovery Workshops) with a porcine intestine membrane. Fully engorged females were selected and provided access to a 10% sugar solution in an incubation chamber with conditions similar for insect rearing. At fourteen days after the blood meal, 25 mosquitoes were dissected into head/thorax and abdomen halves; this resulted in two halves for each mosquito. Each tissue portion was triturated in 500 μl of maintenance medium as described above and the virus load quantified by RT-qPCR. Three oral infection replicates were performed for each virus-mosquito species combination.

### Saliva collection

Five three to five day-old *Ae*. *malayensis* females were orally infected with 1x10^7^ pfu/ml of DENV and 1x10^6^ pfu/ml of CHIKV as described above and held for 14 days. The probosces of six infected *Ae*. *malayensis* were each inserted into a 10 μl pipette tip containing 6 μl of a 1:1 solution of 15% sucrose and heat-inactivated FCS. The mosquitoes were allowed to expectorate for 30 minutes, after which they were triturated as in the oral infection experiment and quantified for the virus load by RT-qPCR. 0.2 μl of the saliva-containing solution was inoculated intrathoracically into five uninfected *Ae*. *aegypti* female mosquitoes aged 3–5 days old using NanoJect II injector (Drummond). Seven days after saliva inoculation, each mosquito was triturated in 500μl of maintenance medium as described above and their virus load was quantified using RT-qPCR.

### Quantification of dengue and chikungunya virus load

Triturated mosquitoes or tissues were centrifuged and 400 μl of supernatant was used to extract viral RNA (QIAamp Viral RNA Mini Kit, Qiagen). DENV or CHIKV genome copies were quantified using a one-step RT-qPCR with iTaq Universal probe kit (BioRad) and primers and probes targeting the envelope (DENV) [37] and nsP1 (CHIKV) genes [38]. The 25 μl reaction mix contained 1 μM of forward and reverse primer, 0.125 μM of probe and 4 μl of RNA extract. Thermal profile started at 50°C for 10 min, 95°C for 1 min and 40 cycles of 95°C for 10 sec and 60°C for 15 sec.

An absolute standard curve was generated by amplifying fragments containing the qPCR targets (one fragment for each virus) using forward primers tagged with a T7 promoter; for DENV we used 5'-TAATACGACTCACTATAGGGCAGGATAAGAGGTTCGTCTG-3' and 5'-TTGACTCTTGTTTATCCGCT-3'; for CHIKV we used 5'-TAATACGACTCACTATAGGGTAGAGCGTTGACCCTACTGA-3' and 5'-AAGGATGCCGGTCATTTGAT-3'. The fragments were reverse transcribed using a MegaScript T7 transcription kit (Ambion) and the total amount of RNA was quantified using a Nanodrop (ThermoScientific) to estimate copy number.

### Screening of field-collected mosquitoes for viruses

Mosquitoes were collected weekly from June-August, 2013 in Sembawang using CDC traps (Bioquip), BG Sentinel Traps (Biogents), and Mosquito Magnet Mosquito trapper (Mosquito Magnet) that were baited with dry ice. Samples were held at 4°C before being brought to Duke-NUS for identification on a chill table. Mosquitoes were pooled by species and collection week. *Aedes albopictus* and *Ae*. *malayensis* pools were homogenized in 500 μl of maintenance medium as described above. RNA was extracted with a QIAamp Viral RNA Mini Kit (Qiagen), cDNA was generated with Superscript II according to manufacturer’s instructions and screened for flaviviruses with previously described primers (FU1 and cFD3) and protocol [39]. Chikungunya was screened for with a previously described pan-alphavirus RT-PCR [40]. Positive and negative controls were used in each reaction.

### Statistical analysis

We conducted three-way mixed-effect ANCOVA to test the impact of transect site, distance along the transect from the forest edge, and collection date (random variable) on the number of *Ae*. *albopictus* and *Ae*. *malayensis*. Where appropriate, we employed linear regression to study the impact of distance for each individual transect. A one-way ANOVA was used to determine the effect of mosquito species on infection and dissemination rates. We estimated the influence of mosquito species and tissue (abdomen or thorax) on viral genome copy by conducting a two-way ANOVA with tissue nested into species. Tissue was nested into species to estimate variation between tissues within the same species. Viral genome copies were log-transformed to fit a normal distribution. Post-hoc analysis were conducted using Tukey's test. All tests were calculated using Systat 13.0 software (SYSTAT).

## Results

### *Aedes albopictus and Aedes malayensis* are present in forested and open areas of city parks

**Fig 2:**
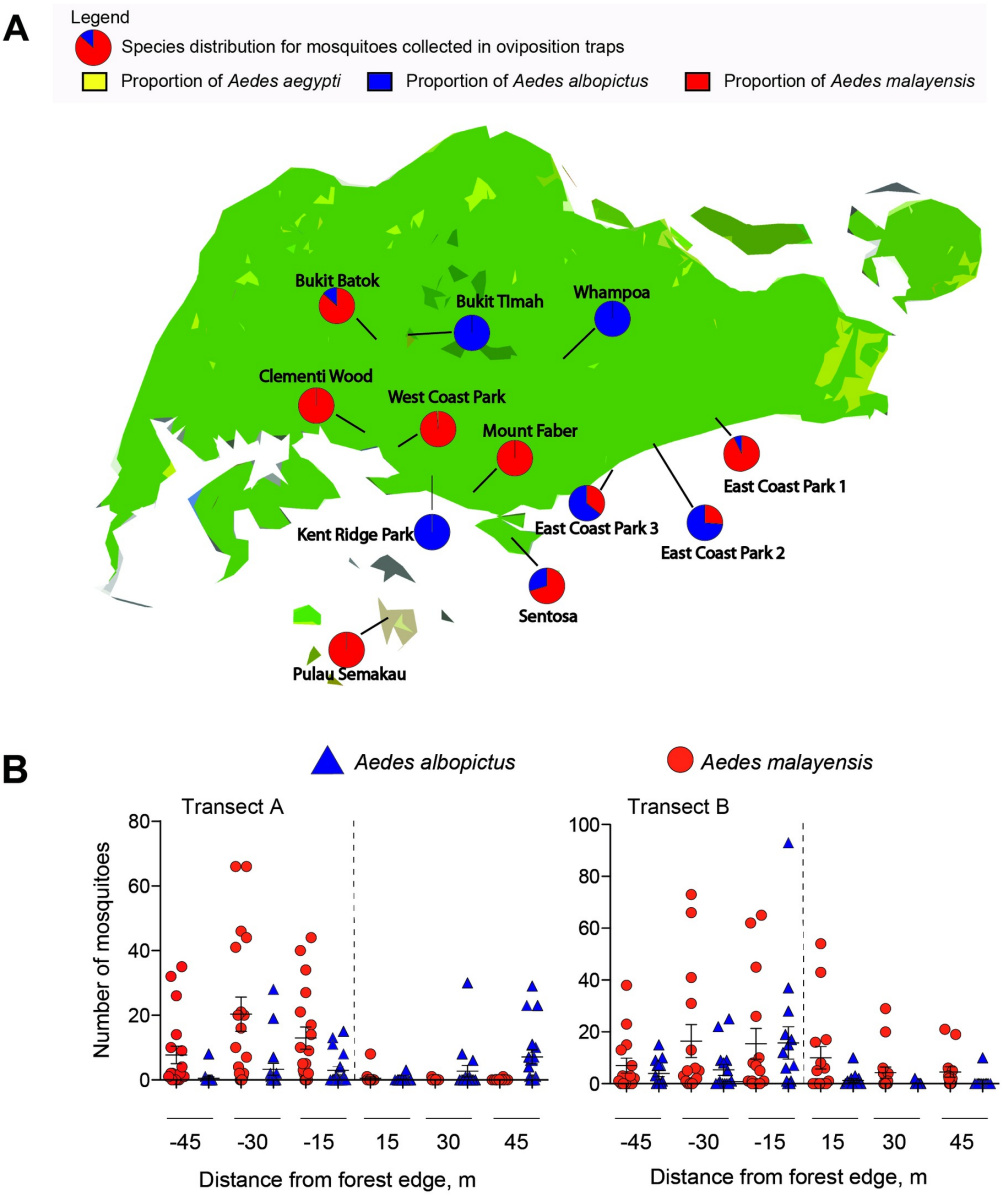
Distribution of *Aedes albopictus* and *Aedes malayensis* in urban parks of Singapore. (A) Proportion of *Ae*. *malayensis*, *Ae*. *albopictus* and *Ae*. *aegypti* reared from eggs collected in oviposition traps distributed in 12 urban parks. (B) Distribution of *Ae*. *malayensis* and *Ae*. *albopictus* reared from eggs collected in oviposition traps distributed across transects that spanned forested to open-air habitat at two sites in East Coast Park Singapore.

To identify sylvatic vectors, we sampled for container-inhabiting *Aedes* mosquitoes in the forested urban parks of Singapore, located in population-dense areas, primarily comprised of high-rise apartments. From the oviposition traps set in 12 forested areas and collected from 2013 to 2015, *Ae*. *albopictus* and *Ae*. *malayensis* comprised almost the entire collection from the oviposition traps set in 12 forested areas with only two *Ae*. *aegypti* were identified (S1 Table). *Aedes malayensis* was the only species detected at three sites, while *Ae*. *albopictus* was the only container-inhabiting species detected in three other sites (Fig 2A). Both species were detected in six sites, and more *Ae*. *malayensis* eggs were collected in four of these sites (S1 Table).

We then determined whether *Ae*. *malayensis* and *Ae*. *albopictus* could be detected in open-air grassy sites of city parks. We collected mosquito eggs from oviposition traps set along two transects from within a forest patch to a grassy open-air area in the most visited park in Singapore [32], East Coast Park. Over the two month period, 8,581 eggs were collected, from which 25.84% were reared to adults, resulting in 1,502 *Ae*. *malayensis* and 716 *Ae*. *albopictus* (S2 Table). Eggs from both species were oviposited in the forested and open areas (Fig 2B), but the effect of distance from the forest edge varied between the two transects for each species (S3 Table). There were more *Ae*. *malayensis* in the forested area of transect A, while there was no difference for transect B (linear regression; transect A: df = 1, 86; p = 0.037; transect B: df = 1, 105; p = 0.066). *Aedes albopictus* distribution was different as mosquitoes were more abundant in the forested area of transect B, but no difference was witnessed between open and forested areas in transect A (transect A: df = 1, 72; p = 0.203; transect B: df = 1, 88; p < 0.001). Together, these results demonstrate that *Ae*. *albopictus* and *Ae*. *malayensis* are present in areas of city parks frequented by humans.

### *Aedes albopictus and Ae*. *malayensis* from Singapore are highly competent vectors for sympatric DENV-2 and CHIKV

**Fig 3:**
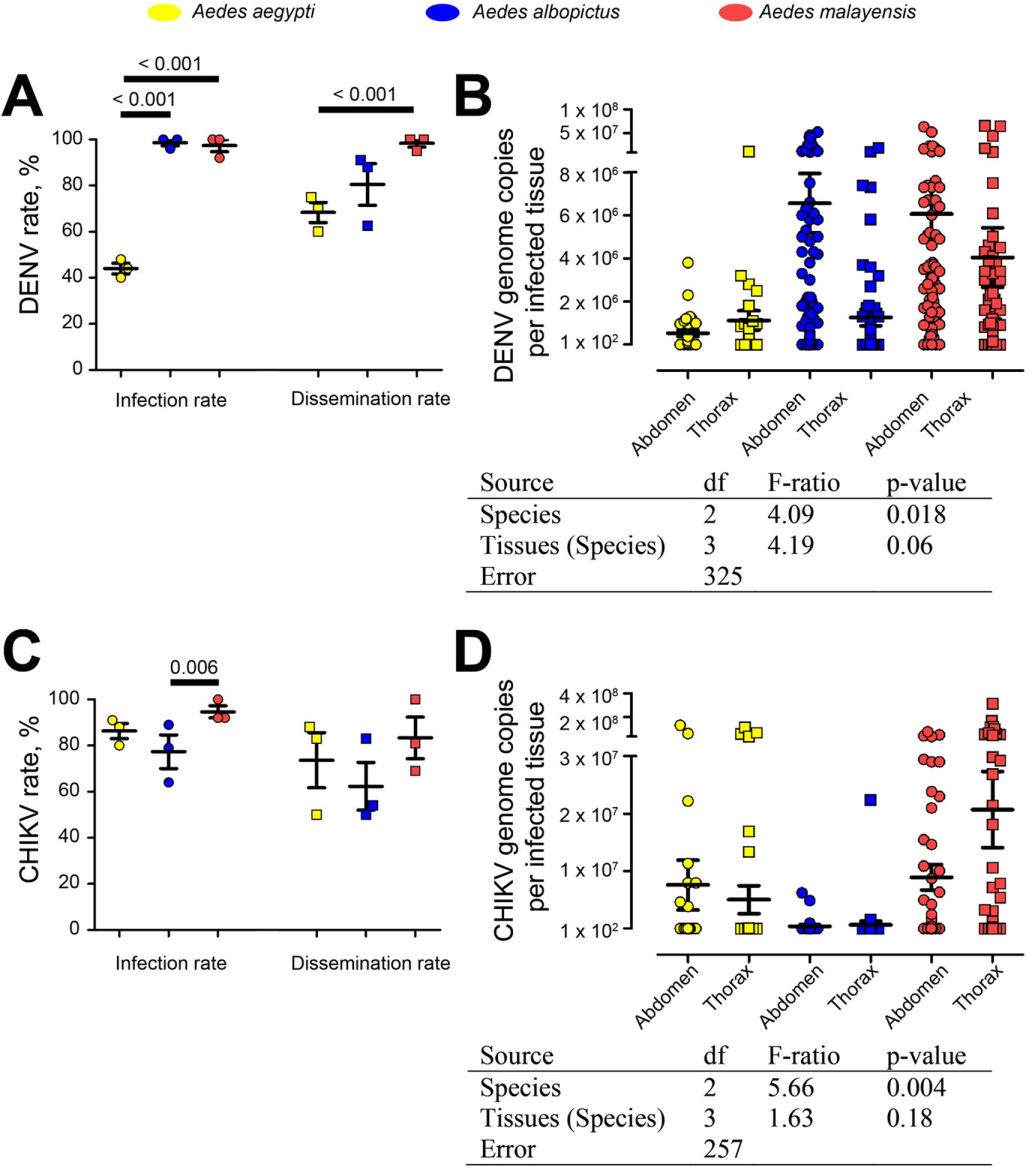
Infection and dissemination abilities for DENV and CHIKV in *Ae*. *aegypti*, *Ae*. *albopictus* and *Ae*. *malayensis*. Mosquitoes were orally infected with the virus and analyzed after 14 days. (A) Infection and dissemination rates for DENV. (B) DENV genome copies per infected tissue in abdomen and thorax. (C) Infection and dissemination rates for CHIKV. (D) CHIKV genome copies per infected tissue in abdomen and thorax. (A and C) Each point represents an independent repeat of 25 mosquitoes. Bars show mean ± s.e.m. T-test significant differences between mosquito species are shown. (B and D). Table below indicates the results from a two-ANOVA testing the impact of species and tissues on viral genome copies per infected tissues.

To determine the vector competence of *Ae*. *albopictus* and *Ae*. *malayensis*, we orally infected the two species with sympatric isolates of DENV serotype 2 and CHIKV. Orally challenged *Ae*. *aegypti* were used as controls. At 14 days post-infection, mosquitoes were separated in two halves corresponding to the abdomen and head/thorax, and the virus was quantified in each section. Detection of virus in the thorax was indicative of dissemination from the midgut to the salivary glands, which are present in the thoracic compartment and represent the source organ for transmission via saliva during biting. DENV infection rates were the highest in *Ae*. *albopictus* and *Ae*. *malayensis*, reaching 100% in two of the three biological repeats, while *Ae*. *aegypti* infection rate was significantly lower than both species with an average of 44% (Fig 3A). Total viral genome copies from both body parts was lower in *Ae*. *aegypti* than in the two other species (Fig 3B; S4A Table). Dissemination rates in *Ae*. *malayensis* reached 100% in two repeats and was significantly higher than in *Ae*. *aegypti* and higher than in *Ae*. *albopictus* (Fig 3A). Viral genome copies per tissue was significantly higher in thoraxes of *Ae*. *albopictus* and *Ae*. *malayensis* than for *Ae*. *aegypti* (Fig 3B).CHIKV infection rates were significantly higher in *Ae*. *malayensis* than in *Ae*. *albopictus* and higher than in *Ae*. *aegypti*, although average infection was above 75% for all three species (Fig 3C). While not significant, average dissemination rate and total viral genome copies in both abdomen and thorax parts were also higher for *Ae*. *malayensis* than for the two other species (Fig 3C and 3D; S4B Table).

To detect arboviruses in field-caught *Ae*. *malayensis* and *Ae*. *albopictus*, we collected a total of 2,432 female *Ae*. *albopictus* and 392 female *Ae*. *malayensis* from 132 traps nights. Each species was collected in each trap type (S6 Table). There were 15 *Ae*. *malayensis* and 54 *Ae*. *albopictus* pools tested for flaviviruses and alphaviruses. All pools were negative for both virus families (S7 Table).

To confirm *Ae*. *malayensis* transmission capacity, we quantified viral genome copies in *Ae*. *aegypti* injected with saliva from orally infected *Ae*. *malayensis*. DENV-infected saliva was infectious to a majority of mosquitoes (Table 1; S5A Table). While one saliva extract did not lead to infection, the four other extracts contained enough infectious particles to infect at least 40% of the saliva-inoculated mosquitoes. All CHIKV-infected saliva contained enough virus to infect mosquitoes (Table 1; S5B Table). Two saliva extracts showed high infectivity as they led to CHIKV infection in all the five saliva-inoculated mosquitoes. Inoculation of the other saliva extracts infected between 40 and 80% of the mosquitoes.

**Table 1:** Infection rate and viral genome copies per infected *Ae*. *malayensis* 14 days after injection of saliva collected from DENV- or CHIKV-infected *Ae*. *malayensis*. nd; not detected.

	DENV			CHIKV		
	Genome copies per mosquito		Infection rate (n / N)	Genome copies per mosquito		Infection rate (n / N)
Repeat	Saliva-collected mosquitoes	Saliva-injected mosquitoes		Saliva-collected mosquitoes	Saliva-injected mosquitoes	
1	4.4 x 10^6^	2.4 x 10^4^	3 / 5	1.3 x 10^8^	1.4 x 10^8^	5 / 5
2	1.8 x 10^7^	1.1 x 10^3^	3 / 5	1.2 x 10^8^	3.1 x 10^8^	2 / 5
3	4.5 x 10^6^	1.6 x 10^7^	5 / 5	1.8 x 10^8^	8.5 x 10^2^	2 / 5
4	9.1 x 10^6^	1.0 x 10^5^	3 / 5	5.2 x 10^7^	7.4 x 10^7^	4 / 5
5	6.6 x 10^6^	nd	0 / 5	1.4 x 10^8^	1.1 x 10^8^	5 / 5
Average		4.0 x 10^6^	14 / 25		1.3 x 10^8^	18 / 25

## Discussion

Here, we addressed the question of whether outdoor peridomestic mosquito species can participate in the epidemiology of dengue and chikungunya in a highly populated and developed city such as Singapore. Our study revealed the high prevalence of *Ae*. *albopictus* and *Ae*. *malayensis* in open-air spaces of forested areas, their distribution across Singapore and their high vector competence for DENV and CHIKV.

Confronted with a global rise in the incidence of dengue and chikungunya cases in cities [41], public health authorities need to question the dogma of current vector control approaches that focus on domestic *Aedes* populations. Changes in world demography and epidemiology has altered the vector control framework [15, 41]. Increase in population density and in global transportation augmented mosquito-human interactions and arbovirus introduction frequency. Interestingly, fine-scale correlation between a reduced *Aedes* house index and lower dengue burden has seldom been observed [42, 43]. Instead, risk of infection is independent of distance from home and determined by human movement into arboviral foci of infection [44, 45]. High risk transmission areas often have narrow spatial-temporal windows [46, 47], likely because mosquitoes are short-lived (less than 3 weeks) and rarely disperse further than 100m [48, 49]. Identification of sites, for example schools, temples or open-air places [33, 50], and pro-active interventions will enhance the efficacy of vector control. In this transmission context, our findings indicate that peridomestic areas may present a risk for infection.

A combination of biological features determines the vector status of mosquito species, two of which are especially important [51]. The first is their susceptibility to infection, enabling replication of the virus to high titers throughout the mosquito body and particularly in the salivary glands, thus facilitating transmission during subsequent blood feeding [52]. Importantly, vector competence can be affected by intra-species mosquito diversity and virus origin [53, 54]. We therefore conducted a vector competence study with sympatric mosquitoes and viruses. A second important characteristic responsible for the vector status is the proclivity to bite humans, which depends on its anthropophily and presence in human populated areas, such as open-air parks where we identified the two sylvatic species for which we characterized vector competence.

*Aedes albopictus* is known to be susceptible to DENV and CHIKV infection [51, 55, 56], and in some instances, was found to be more competent than *Ae*. *aegypti* [57, 58], as this was the case in our study. Our results demonstrated a higher DENV infection rate for *Ae*. *albopictus* than for *Ae*. *aegypti*. Of note, the relatively low infection rates for *Ae*. *aegypti* were comparable to previous experiments [58]. The global chikungunya outbreak initiated in 2004 was caused by enhanced transmission efficiency by *Ae*. *albopictus* following a mutation in the E1 protein of the virus [9, 59], although cross genetic interactions between mosquito and virus strains can affect transmission [60]. Despite the mutation absence from our local isolate, infection and dissemination rates in *Ae*. *albopictus* were not significantly different to the ones in *Ae*. *aegypti*, indicating *Ae*. *albopictus* from Singapore is likely capable of transmitting both CHIKV strains, similarly to *Ae*. *aegypti* [61]. Both CHIKV strains are now globally distributed and could be introduced to Singapore [62], thereby increasing the risk of transmission by *Ae*. *albopictus*. Moreover, *Ae*. *albopictus* has been shown to prefer to bite humans even in the presence of other vertebrates [7]. *Aedes albopictus* was incriminated as the primary vector in epidemics of dengue and chikungunya in central Africa [63], China [64] and Mediterranean Europe [65, 66]. Interestingly, an epidemiological study in a Japanese city showed that more than 80% of dengue patients during an epidemic visited a city park where *Ae*. *albopictus* is present [67].

*Aedes malayensis* (Colless 1962) is a relatively understudied species with a wide distribution in Southeast Asia [68]. Its presence has been recorded in Taiwan, Vietnam, Cambodia, Peninsular Malaysia, the Andaman and Nicobar Islands of India and across several regions in Thailand [34, 69, 70]. In our study, differences in *Ae*. *malayensis* prevalence between sampling sites may reflect variable microhabitat characteristics [71]. Sites with a majority of *Ae*. *malayensis* were mainly young secondary forests, whereas sites devoid of *Ae*. *malayensis* were a mixture of primary and secondary forests. *Aedes malayensis* vector competence has not been fully characterized for DENV [58] and is undocumented for CHIKV. A previous study showed the susceptibility of *Ae*. *malayensis* to all 4 DENV serotypes after oral infection [58] but did not determine the presence of virus in the saliva. Our data clearly established the high susceptibility of *Ae*. *malayensis* and validated its transmission capacity for both DENV-2 and CHIKV. Detection of virus in field-caught vectors is important evidence of potential vector capacity in natural settings. We did not detect DENV or CHIKV in our field-caught *Ae*. *malayensis*. However, virus detection in field-caught vectors is challenging. For instance, a previous study failed to detect DENV in *Ae*. *aegypti* from the Cape Verde Island where the four DENV serotypes are endemic [72]. Importantly, *Ae*. *malayensis* have been shown to bite humans [68, 69] even though they oviposit outside of domiciles [68, 69, 73, 74]. Altogether, these results establish for the first time the vector competence of *Ae*. *malayensis* for sympatric DENV-2 and CHIKV and warrants additional study to determine its role as a vector in Southeast Asia. There are a number of other *Stegomyia* species in Southeast Asia that may be capable of transmitting DENV and CHIKV and should be investigated as well [2, 75–78]. Such peridomestic vectors have been involved in transmitting epidemics of dengue and chikungunya [76, 79].

Our study has some limitations in incriminating *Ae*. *malayensis* and *Ae*. *albopictus* as important vectors in Singapore. Although vector biting rate can elucidate the involvement of vectors in pathogen transmission, we did not measure this parameter. The absence of prophylaxis or curative agents against these viruses does not warrant ethical clearance for human landing rate studies. Alternatively to our hypothesis that peridomestic *Aedes* transmission is a cause of recurrent arbovirus outbreaks in Singapore and generally in cities, frequent introductions of viruses from neighboring endemic countries may play a role [41]. Further molecular epidemiology studies are needed to determine the relative importance of virus introduction and peridomestic transmission.

Singapore represents a particular case in Southeast Asia and may illustrate the long-term frailties of the current vector control approach. We should learn from this example to support the continued monitoring of the efficacy of vector management. One of the richest countries (GDP per capita) in the world [80], Singapore is primarily comprised of high-rise buildings [81] equipped with architectural features that reduce potential larval habitats. This city-state can afford a permanent evidence-based integrated vector control programme widely regarded as a worldwide success [42]. The *Aedes* house index has been maintained at 1–2% for the past 45 years [18], which is well below the 4% threshold defined as an epidemic alert [82]. Yet, the low dengue and chikungunya incidence could not be sustained for more than 30 years [18, 83]. Here, we propose that the presence of highly competent vectors in city parks and forested areas may be contributing to transmission and maintenance of the viruses. Maintenance of low infection rates in few areas is enough to ensure the persistence of arboviruses in large cities and to initiate an outbreak when conditions are favorable [84]. The presence of non-human primates, such as macaques in Singapore, coupled with competent zoonotic vectors may also provide alternative reservoirs as refugia for arboviruses [85, 86]. Risk of infection in parks is increased by the low herd immunity of the Singaporean population [19, 29], the potential presence of asymptomatically infected people or those prior to clinical disease onset who are infectious to mosquitoes [87], and wearing clothes that expose skin to mosquito biting. Furthermore, the high selection pressure [88] on arbovirus transmission due to the overall low domestic vector population may select for more transmissible virus strains. Although further studies, including fine-scale tracking of patients and molecular epidemiology, are required to confirm localization of the foci of infection, our results strongly support the inclusion of peridomestic areas in the scope of vector control and surveillance programmes.

## Supporting information

S1 Fig Morphological differences between *Ae*. *malayensis* (A) and *Ae*. *albopictus* (B). The primary differences are the suprealar scale patch extending to the scutellum in *Ae*. *malayensis* (i), the scalloped pattern of abdominal tergites (IV-VI) on *Ae*. *malayensis* (ii), and the absence of scales from the subspiracular area in *Ae*. *malayensis* (iii). Images credited to Nicky Bay.
(TIF)

S1 Table Ovitrap collection data and reared mosquito species for 12 trapping sites in Singapore. (XLSX)

S2 Table Total numbers of eggs collected and mosquitoes reared from Transect A and B in East Coast Park, Singapore, for the patch fidelity and oviposition experiments.
(XLSX)

S3 Table Results from ANCOVA of oviposition preference for *Ae*. *malayensis* and *Ae*. *albopictus* along two transects that cover forested and open areas in East Cost Park, Singapore. (XLSX)

S4 Table Viral genome copy number in abdomen and thorax of *Ae*. *aegypti*, *Ae*. *albopictus* and *Ae*. *malayensis* at 14 days post oral infection with (A) DENV or (B) CHIKV. nd: not detected.
(XLSX)

S5 Table Infection rate and viral genome copy number for *Ae*. *malayensis* injected with saliva collected from DENV- (A) or CHIKV- (B) infected *Ae*. *malayensis*. nd: not detected.
(XLSX)

S6 Table Mosquito collection data from four transects in Sembawang, Singapore in 2013 using CDC traps, BG Sentinel traps, and Mosquito Magnet traps. (XLSX)

S7 Table Flavivirus and alphavirus PCR results for *Aedes malayensis* and *Aedes albopictus* pools collected in Sembawang, Singapore. (XLSX)
