# Supplementary figures and images for: Peridomestic Aedes malayensis and Aedes albopictus are capable vectors of arboviruses in cities
Source: PLoS Negl Trop Dis. 2017 Jun 26;11(6):e0005667. doi: 10.1371/journal.pntd.0005667 (PMC5501678; doi:10.1371/journal.pntd.0005667)

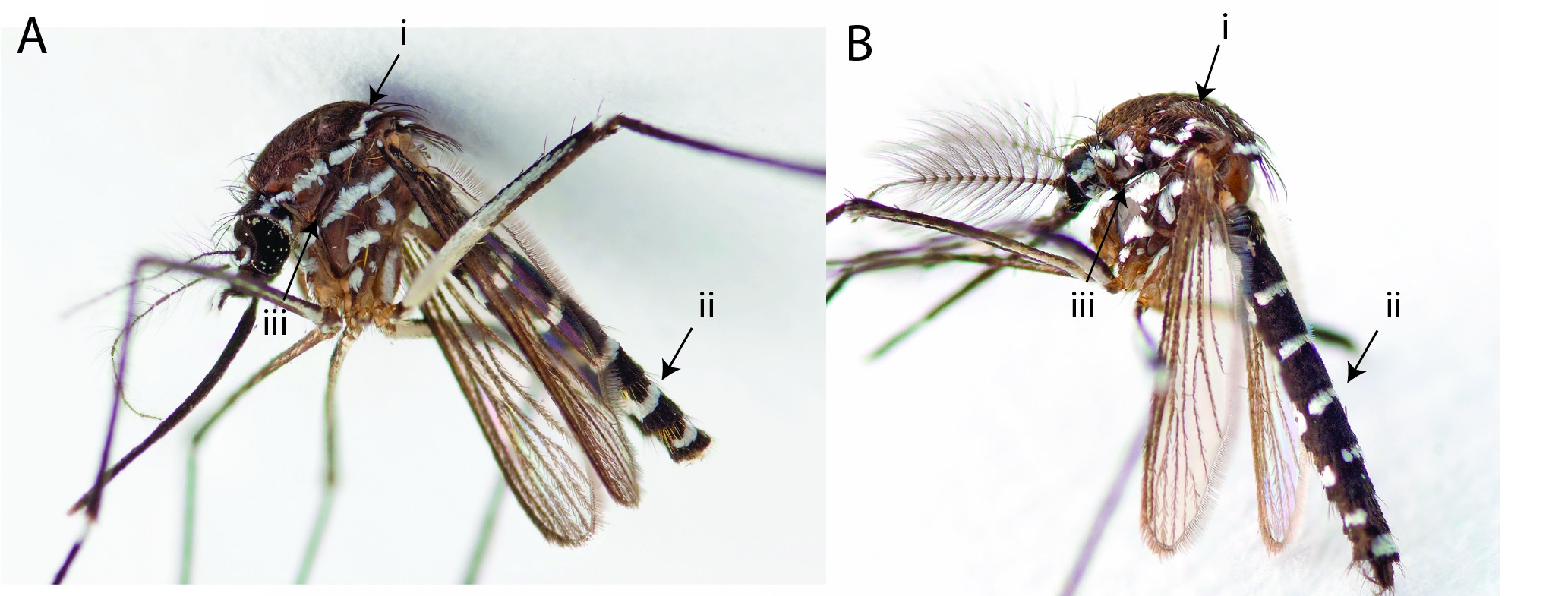

Supplement: S1 Fig — Morphological differences between Ae. malayensis (A) and Ae. albopictus (B). The primary differences are the suprealar scale patch extending to the scutellum in Ae. malayensis (i), the scalloped pattern of abdominal tergites (IV-VI) on Ae. malayensis (ii), and the absence of scales from the subspiracular area in Ae. malayensis (iii). Images credited to Nicky Bay. (TIF) [file pntd.0005667.s001.tif]
